# The Psychometric Properties of the Original Version of Assessment of Time Management Skills (ATMS)

**DOI:** 10.1155/2022/6949102

**Published:** 2022-02-03

**Authors:** Afsaneh Roshanay, Gunnel Janeslätt, Kajsa Lidström-Holmqvist, Suzanne White, Marie Holmefur

**Affiliations:** ^1^Department of Public Health and Caring Sciences, Uppsala University, Uppsala, Sweden; ^2^Department of Public Health and Caring Sciences, Disability and Habilitation, Uppsala University and Center for Clinical Research in Dalarna, Region Dalarna, Sweden; ^3^University Health Care Research Center, Faculty of Medicine and Health, Örebro University, Örebro, Sweden; ^4^State University of New York, Downstate Medical Center, Brooklyn New York, USA; ^5^School of Health Sciences, Faculty of Medicine and Health, Örebro University, Örebro, Sweden

## Abstract

**Background:**

To perform daily activities, time management and organizational skills are essential and therefore also important intervention focus in occupational therapy. To guide and evaluate intervention, valid and reliable instruments that measure time management and organization skills are necessary. The aim of this study was to evaluate the psychometric properties of the Assessment of Time Management Skills (ATMS).

**Methods:**

Eligible participants were volunteer adults from the general population who were aged between 18 and 65 years, had a good understanding of English, and were not currently involved in any training or education to improve time management. The ATMS was filled out as a computer-administered survey. Rasch measurement analysis was used to evaluate the validity and aspects of reliability of the ATMS.

**Results:**

In total, 241 adults (112 male and 129 female, mean age = 40) participated. The analysis of principal components of residuals (PCA) and the item goodness-of-fit indicated that the 30-item scale does not measure only one single trait. Three subscales, time management (11 items), organization and planning (11 items), and regulation of emotion (5 items), were detected. One item each on the 11-item subscale showed misfit, but they were kept due to high relevance. All three subscales showed excellent results on analyses of PCA, local independence, and reliability. *Conclusions and Relevance*. ATMS can provide valid measures of time management, organization and planning skills, and regulation of emotion in a general population and presumably also in a population with cognitive impairment. The measure is useful for occupational therapists in assessing patients' strengths and barriers in time management skills. It can also be useful in identifying the necessity of training time management skills, to guide OT intervention programs and to evaluate interventions. *What This Article Adds*. Knowledge about the psychometric properties and usefulness of the ATMS in English-speaking countries.

## 1. Introduction

Time management is considered to be a key intervention component to enhance performance and satisfaction with daily occupations [1–3]. In order to plan and evaluate rehabilitation, valid and reliable instruments adjusted for the specific target group and specific culture where they will be used are essential. However, instruments are constructed on different definitions of time management and consequently do not represent similar operationalizations of the time management construct [4].

Time management is a component of executive functions and has been defined as “*the mental functions of ordering events in chronological sequence, allocating amounts of time to events and activities*” (b1642, p.57) [5]. Time management also includes the more complex behavior of planning activities in relation to the actual time available in daily life [5]. Time management skills are closely related to organization and planning skills [6]. In the ICF, organization and planning are described as the “*mental functions of coordinating parts into a whole, of systematizing; the mental function involved in developing a method of proceeding or acting*” (b1641, p.57) [5].

Executive functioning including time management and organization and planning skills is typically affected in persons suffering from mental disorders, substance abuse disorders, neurodevelopmental disorders, or intellectual disabilities, among others. In people with neurodevelopmental disorders, signs of ineffective time management include difficulties to complete tasks on time and procrastination [7], and executive dysfunction might be related to difficulties in emotion regulation [8]. Emotion regulation is defined as a process by which emotions are controlled, how intensely and for how long they are experienced [9], and how they are expressed [5, 10].

Because of the impact that time management and related skills have in daily life, they are an important focus of rehabilitation for individuals with cognitive restrictions. Hence, there is a need to measure time management skills and to determine which aspects an instrument measures. To our knowledge, there are few validated instruments available in the field of time management. In addition, they have different focuses, or aims, such as capturing feelings related to different time perspectives [11], estimation of time [12], or guiding intervention with time assistive devices [13]. The Assessment of Time Management Skills (ATMS) questionnaire was developed in the United States as a means of evaluating time management skills. The target group was individuals with serious mental illness and coexisting substance-related disorders [14]. The ATMS is a self-reported 30-item questionnaire aimed at measuring how and how much people adapt their behavior to manage time in daily life [14]. Recently, a Swedish version of the ATMS (ATMS-S) was developed by our research group [15], by translating and adapting it culturally and conceptually to Swedish society. The instrument was also adapted to an expanded target group including individuals with neurodevelopmental or mild intellectual disability. Results from a construct validity evaluation using Rasch analysis in persons with and without disabilities (*n* = 94 and *n* = 144, respectively) showed that the ATMS-S (when reduced to 27 items) measures three constructs (1) time management, (2) organization and planning, and (3) regulation of emotions related to time management [15]; however, construct validity has not been evaluated in the original English version. The ATMS-S has been used to evaluate intervention in a pilot study in Sweden [2] and proved useful to detect changes in individuals with cognitive impairments. For the ATMS to be of further use in English language contexts, its construct validity needs to be assessed. The aim of this study was to evaluate the construct validity and reliability of the ATMS.

## 2. Materials and Methods

The sample in the current study was the same as in White et al. (2013). In 2009, a sample of 241 adults from the general population was recruited by a quality-assured sampling service run by the company Market Tools' True Sample, which conducted screening of participants with regard to their credibility. Criteria for inclusion were age 18 to 65 years, understanding of English, and no current involvement in any training to improve time management.

### 2.1. Assessment of Time Management Skills (ATMS)

ATMS is a self-reported 30-item questionnaire designed to measure how the users actively benefit from cognitive strategies and adaptations such as using calendars or lists for planning tasks in daily life and how aware users are of their time management skills. Each item is a statement describing time-related daily life situations or opinions of one's own time management skills. A four-graded response scale, from none of the time (1) to all of the time (4), is used. Psychometric properties of ATMS evaluating the internal consistency (*α* = 0.86) in a norm population (*n* = 241) and test-retest reliability (*r* = 0.89) assessed by the Pearson correlation coefficient are presented elsewhere [14].

### 2.2. Data Collection

The questionnaire was distributed as a computer-administered test located at the online survey tool Zoomerang. The online survey was introduced with a few study-specific questions on demographic information: age, sex, marital status, race, and level of education, and it was filled out anonymously, i.e., Market Tools' staff, or the researchers did not obtain identifying data at any time. Participants could answer the survey by logging onto the Zoomerang website, and they received $10 via Zoomerang upon completion of the survey. In order to maintain confidentiality, the company received the answers in a numbered fashion. Once the participants had completed the questionnaire, Market Tools provided the researchers an Excel spreadsheet with the participants' answers matched to their numbers.

### 2.3. Data Analysis

To evaluate the construct validity and reliability of the ATMS, Rasch analysis, a widely used method for creating and evaluating instruments in the social and health sciences [16], was conducted with Winsteps software version 3.80.1 [17]. Descriptive data were summarized with IBM SPSS (Statistical Package of the Social Sciences) statistical software, version 24.0.

First, the entire original scale of 30 items was analyzed, followed by separate analyses of the three subscales found by Janeslätt et al. [15]. The same six-step procedure, described below, was used for each analysis. In all analyses, the Rating Scale Model was used, based on the assumption that all items shared a common rating scale structure [18].

- The structural integrity of the scale was assessed by examining rating scale category functioning to ensure the absence of disordered thresholds. The criteria for a category to be interpreted as well-functioning were a category count of at least 10 responses per category and an outfit mean square (MNSQ) of <2, and that average measures and item thresholds increased monotonically for each step up on the scale [19]. The thresholds should be between 1.4 and 5 logits to distinguish meaningfully between categories [19]

Unidimensionality is a fundamental requirement in Rasch models and was evaluated in steps B and C

(B) A goodness of fit analysis indicates how well each item fits within the underlying test construct [20]. Criteria for item fit were infit MNSQ less than 1.4 along with a *z*-standardized (ZSTD) value between -2 and +2 [18]
(C) A principal component analysis (PCA) of the residuals was conducted. In the PCA, there should not be any meaningful patterns in the residuals except from the construct intended to measure. The unidimensionality of the measurement is considered to be poor if the Eigenvalue of PCA for the first contrast is higher than 2 [18]. Misfitting items need to be investigated in more detail and may need to be reworded or removed
(D) Local independency is another important requirement which means that the response to one item should not be dependent on the response to another item [21]. Local independency was assessed by interitem residual correlation, where a correlation below 0.3 was considered sufficient [22, 23]
(E) Targeting, the location of the item difficulty relative to the respondents' ability, was investigated by inspecting the item-person map and by the difference between item and person logit means, which should not exceed 0.5 logits
(F) Reliability was measured by person and item reliability coefficients. The distinguishable number of levels of time management (strata) was calculated by first extracting the person separation index (*G*) from the person reliability coefficient and then using the formula (4*G* + 1)/3 [24]. The same way number of item strata was calculated, which shows to what extent the scale can measure the full difficulty range of the construct measured. To be considered acceptable, there should be at least 2.0 strata and associated reliability coefficients should be >0.8 [25]
(G) To test the hypothesis that items do not function differently depending on gender or education level, Differential Item Functioning (DIF) was investigated [18]. Criteria for DIF were a contrast of >0.5 logits together with significance in the Mantel test [26]. The size of DIF must be large enough to reflect a substantial difference in performance.

In cases of any changes, analyses were rerun and decisions about revising or removing items were made by taking into account information from all steps of analysis. For the final subscales, the logit measures were transformed to a 0-100 ATMS unit scale.

## 3. Results

In the current study, 241 adults from the general population participated: 112 males and 129 females. Demographic data on participants are presented in Table 1.

In the first analysis of the ATMS scale including 30 items, there were >10 responses per category, the category and threshold measures did advance monotonically, and the category outfit MNSQ was between 0.49 and 1.81. Four items—4, 27, 28, and 10—displayed misfit (infit MNSQ and *z* value = 1.82, 8.1; 1.48, 4.7; 1.77, 7.2; and 1.91, 8.1, respectively). In the PCA, the Eigenvalue of the first construct was 4.5, indicating that the 30 items probably represented more than one dimension. The largest interitem correlation was 0.69 and indicated local dependency. Inspection of the person-item map showed good targeting between persons and items. The person and item reliability coefficients were 0.83 and 0.98, respectively, and the scale could distinguish between two different ability strata. The results of person and item reliability indicated that ATMS items were highly consistent and replicable, but it was probably not measuring only one single trait according to the PCA of the residuals. Consequently, ATMS items were divided into subscales: time management (11 items), organization and planning (11 items), and regulation of emotion (5 items) based on the Swedish findings [15] in order to explore whether the same structure holds. Items 3, 4, and 27 which illustrated misfit in both the Swedish and English versions were omitted.

### 3.1. Time Management Subscale

The time management subscale (11 items) results showed good psychometric properties. The rating scale was well functioning and fulfilled the criteria, as shown by category and threshold measures advancing monotonically and a category outfit MNSQ of 0.70 to 1.32. In the item goodness-of-fit analysis, one of the 11 items, item 10 (“*I find that even though I want to be on time, I am often late*”), showed misfit (infit MNSQ = 1.48, *z* = 4.7). The Eigenvalue of the first construct in PCA was 2.1, and the largest interitem correlation was 0.20, indicating local independency. The targeting between person and item measures as seen in the person-item map was good, but the mean person ability measure in the sample was 1.45 logits higher than the mean item measure. The item reliability was 0.96 and person reliability was 0.77, and two different ability strata could be distinguished (Table 2). The DIF analysis indicates that there was no evidence to suggest that the time management subscale items functioned differently between males and females or among individuals with different education levels (Table 3). A renewed analysis was conducted without item 10, which had shown misfit. All 10 remaining items fit the model, and all other values presented minor changes (Table 2).

### 3.2. Organization and Planning Subscale

The rating scale for the organization and planning items was considered to be well functioning because it fulfilled the criteria (outfit MNSQ ranged between 0.67 and 1.40). The item fit analysis of the organization and planning subscale (11 items) showed a large misfit on item 28 (“*I wear a watch or carry a cell phone to keep track of the time*”), infit MNSQ = 1.77, *z* = 7.2 (Table 2). The PCA of the 11-item scale showed an Eigenvalue of 2.0 indicating unidimensionality, and the largest interitem correlation was 0.24 indicating local independency. There was good targeting between person and item measures in the organization and planning subscale shown by both the person-item map and a difference of 0.32 logits between person measures and item measures where the person measures had the higher value. In the reliability evaluation, two strata were found and the item and person reliability coefficients were 0.98 and 0.73, respectively. No evidence to suggest that the subscale items functioned differently between males and females or between individuals with different educational levels was found (Table 4). A renewed analysis was conducted without the misfitting item (item 28), and all remaining 10 items fit the model. All other values showed minor changes (Table 2).

### 3.3. Regulation of Emotion Subscale

Also, the regulation of the emotion subscale's rating scale functioned well and fulfilled the criteria. In the item fit analysis, all five items showed good fit (Table 2), and an Eigenvalue of 1.6 was found in the PCA. With the largest interitem correlation being .01, there was no local dependency between responses. The targeting was good, since the mean person measures were 0.02 logits higher than the item average. Person reliability was 0.79 and item reliability was 0.98, and the subscale distinguished between two ability levels. The subscale regulation of emotion functioned equally for males and females and individuals with different levels of education (Table 3). Hence, measurement properties of the regulation of emotion subscale are excellent.

In summary, the analysis resulted in three distinctly different subscales, the time management subscale and the organization and planning subscale, both with 11 items each and the regulation of emotion subscale including 5 items (Tables 2 and 3). Subscale raw scores can be converted to interval-level ATMS unit scores ranging from 0 to 100 via Table 4.

## 4. Discussion

This study is aimed at evaluating the psychometric properties of the original English version of ATMS. The analysis of the person and item reliability of all 30 items indicated that although ATMS items were consistent and replicable, there is evidence that the scale does not measure only one single trait and is therefore less useful as a single scale. However, a renewed analysis dividing ATMS into time management, organization and planning, and regulation of emotion subscales indicated an overall very strong construct validity and reliability, in line with the results found in the analyses of ATMS-S by our research group.

According to the Rasch model, the time management subscale with eleven items showed good measurement properties. Still, item 10 (“*I find that even though I want to be on time, I am often late*”) showed misfit to the model. This item captures a situation often described and easily recognized by people with difficulties in time management skills. Thus, the item was considered a clinically important complement to the other items on the scale. The order of the wording of the phrase possibly creates a challenge, posing an argument for rewording. Since all ten remaining items in the time management subscale fit the model, rewording item 10 might improve the measurement properties. Further, when scrutinizing the items, we find that this subscale contains items on both of the cognitive functions of time management skills, namely, ordering events chronologically and allocating time length to events and activities, as well as the more complex behavior of planning activities in relation to the time available in daily life. Our evaluation showed that the time management subscale does not distinguish between items measuring the cognitive function from items closer to activity and participation. Nevertheless, together, the items relate to both aspects of time management, i.e., time management skills. Both Holmefur et al. [2] and Wingren et al. [27] show that the subscale is also sensitive to change.

In the analyses of the organization and planning subscale (11 items), the PCA indicated unidimensionality despite item 28 (“*I wear a watch or carry a cell phone to keep track of the time*”) showing a large misfit (infit MNSQ = 1.77, *z* = 7.2, Table 2). This item showed a misfit in the Swedish material as well (infit MNSQ = 1.45, *z* = 3.3) [15] and therefore needs to be reflected upon. This item has been reworded. It would be more readily understood to use the visual action “*I look at a watch or a cell phone to keep track of the time*”. The need for skills to use tools to manage time and organization and planning is well established. A systematic review by Gillespie et al. [28] indicates that there is at least moderate evidence that tools (e.g., computers providing reminders) are useful for compensating organization, planning, and time management in both home- and work-related activities, in people with cognitive limitations. Today, a cell phone is a widely spread tool, among many others, which is useful for managing time. In Sweden, 85% of women and 88% of men in the general population use smartphones while persons with disabilities use them to a considerably lesser extent, e.g., ADHD 52%, ID 32%, and bipolar disorder 10% [29]. Not having access to any tool can be a barrier to managing time, so the need for an item to investigate this is warranted. We think that a possible reason for the misfit in item 28 may well be the wording, given that the item starts with a dual question: whether I wear a watch or carry a cell phone. How would people answer if they only use one of them? The important issue here is rather the need of keeping track of time and if they use tools for that purpose. Thus, it is recommended to alter the phrasing of this item. After revising item 28, the subscale can likely assess organization and planning in a more valid and reliable way.

It is notable that although there is no WHO definition of organization and planning on activity and participation level, the items in this subscale seem to be measuring organization and planning in daily life (i.e., activity and participation). Possibly what is measured in the subscale can be an aspect of “managing daily routine” in the ICF (Code d2301 [30]), but using such a wide definition would not be helpful neither clinically nor in research.

The three subscales of the ATMS can be used by occupational therapists when planning and evaluating interventions for clients related to time and planning in daily life. For some client groups, such as adults with ADHD, emotional dysregulation has shown to be the core symptom causing most functional impairment in daily life [31]. An important contribution of ATMS might be addressing these specific aspects of executive functioning, not only time management skills but also organization and planning and regulation of emotions in daily life situations in a compact questionnaire.

In the evaluation process, clinicians use clinical reasoning when choosing assessments to measure the client's strengths and limitations before setting goals for intervention. In this process, clinicians use research-generated knowledge in combination with professional experience and knowledge of the client's specific situation. ATMS might be a complement to other instruments that measure self-reported time management skills, e.g., the TOPS [12] and the Time-S [13]. Time-S can separate the level of time management in daily life in persons with cognitive problems and is useful for guiding and evaluating interventions with time assistive devices (ibid). The TOPS assesses the perceived ability of organizing daily tasks on time [12]. It has three distinctive factors: (1) daily task performance at a pace appropriate to environmental demands, (2) how daily activities are organized, and (3) how the person responds emotionally to disorganization. ATMS and TOPS are partly overlapping. TOPS is more explanatory as it adds the estimation of pace related to specific daily activities and the time demands of the environment, which can add valuable information about the person's ability to handle time frames. The ATMS is an efficient assessment containing information not only on time management and organization and planning but also on regulation of emotions. As such, it fulfills the aim of evaluating intervention when the goal is to increase time management skills such as in the occupational therapy intervention program Let us Get Organized. The Weekly Calendar Planning Activity (WCPA) is another useful instrument in the assessment of executive functions in the activity of time planning [32]. In the WCPA, the client is challenged with a complex task that requires both focus, impulse control, and the ability to plan ahead that is also often the case in daily life. The WCPA can add and deepen information on executive functioning in the aspects of time planning skills as well as the ability to handle distractions and keep track of time while performing a complex task. Thus, it might be of value to combine different instruments focusing on time management in occupational performance in order to get a thorough evaluation [2, 33].

### 4.1. Methodological Considerations

The original target group for ATMS is individuals who suffer from cognitive limitations as a result of mental disorders and substance abuse. One of the study's inclusion criteria is that participants did not participate in training on time management at the time of data collection. This criterion does not exclude participants with a cognitive disability. It should be considered that cognitive disabilities occur in many groups in society; therefore, it is probable that some participants in this study had cognitive disabilities. Further, in the evaluation of the Swedish version, ATMS-S, it was also found to be useful for persons with neurodevelopmental disorders and/or mild intellectual disabilities.

The results of the current study are in line with the Swedish study, in which 40% of participants were individuals with cognitive limitations due to mental disorders [15]. Thus, it might be assumed that the present results are likely to also be representative of the target groups in English language contexts. A consequence of this is that the less optimal targeting of item difficulty to person ability in the time management subscale is not considered to be a problem with the scale because the sample in this study is expected to score higher than the target population. However, it is important to evaluate the targeting and other psychometric properties in a population with cognitive impairment in an English language context as well.

### 4.2. Implications for Occupational Therapy Practice

The ATMS is useful for occupational therapists to help the patients to identify strengths and barriers in time management skills and how it can affect their daily life. The ATMS can be used to identify the need for training time management skills, to guide OT intervention program, and to evaluate interventions.

- The ATMS can provide valid measures of self-assessed time management skills
- The ATMS contain information not only on time management skills and organization and planning but also on regulation of emotions
- The ATMS can be a complement to other instruments that measure self-reported time management skills

## 5. Conclusions

The results of this study add knowledge about the psychometric properties and usefulness of the ATMS in English-speaking countries. It shows that the ATMS can provide valid measures of self-assessed time management skills in a general population and presumably also in a population with cognitive impairment due to mental or neurodevelopmental disorder.

## Figures and Tables

**Table 1:** Demographic characteristics of the participants, *n* = 241.

Variable	
Age (yrs)	
Range	18-65
Mean (SD)	40 (12)
	*n* (%)
Gender	
Male	112 (46.5)
Female	129 (53.5)
Racial/ethnic background	
Native American	1 (0)
African American	12 (5)
Asian American/Pacific Islander	7 (3)
White (non-Hispanic)	207 (86)
Hispanic	9 (4)
Other	5 (2)
Marital status	
Single (never married)	76 (32)
Married	112 (46)
Cohabiting	6 (2)
Separated	28 (12)
Divorced	1 (1)
Widowed	18 (7)
Education	
Less than high school	10 (4)
High school or equivalent	39 (16)
Vocational or technical training	18 (8)
Some college	78 (32)
Graduated college	71 (29)
Postgraduate education	25 (10)

**Table 2:** Items in each of the three subscales, listed in item difficulty order and with item fit statistics.

Item no.	Item content	Item measure (logits)	MNSQ	ZSTD
Time management subscale				
19	I can correctly estimate the time I need to complete my tasks.	1.25	0.91	-0.1
1	I feel I manage my time well.	0.94	0.82	-2.1
18	I put off things I do not like to do until the very last minute.	0.32	0.98	-0.1
24	I feel confident that I can complete my daily routine.	0.16	0.77	-2.7
20	I learn from my mistakes.	0.02	0.99	0.1
7	I rush to complete my work.	-0.06	1.10	1.1
11	Even if I do not like to do something, I still complete it on time.	-0.20	1.34	3.4
9	I find that I am overwhelmed by my daily routine.	-0.33	1.06	0.7
30	I feel that I do not manage my time well.	-0.36	0.99	-1.0
26	I run out of time before I finish important things.	-0.64	0.73	-3.4
10	**I find that even though I want to be on time, I am often late.**	**-1.09**	**1.44**	**4.5**

Organization & planning subscale				
16	I wait until I feel better before taking on important tasks.	0.91	1.11	1.2
5	I stop and plan out the steps before I start something new.	0.54	0.74	-3.3
6	I plan my daily activities.	0.46	0.64	-4.8
2	I use a calendar or an appointment book as a way of remembering my daily tasks.	0.31	1.23	2.6
13	I clean my workspace before beginning a task.	0.23	1.02	0.3
15	I make to-do lists.	0.19	1.07	0.9
21	I make sure I have a good night's sleep.	0.16	0.93	-0.9
8	I do my most difficult work at the time of day when I have the most energy.	0.15	0.65	-4.9
29	I put my things back where they belong or where I got them from.	-0.66	0.83	-2.2
12	I am not organized in my tasks.	-1.00	1.11	1.3
28	**I wear a watch or carry a mobile phone to keep track of the time.**	**-1.28**	**1.76**	**7.2**

Regulation of emotion subscale				
14	I complete the task on my schedule or appointment book to my satisfaction.	0.98	0.77	-2.7
25	I put in more effort to follow my schedule when I see others keeping up with their schedule.	0.97	1.20	2.3
17	I reward myself for doing a good job.	0.94	0.95	-0.6
23	My mood affects my ability to manage my time.	-0.44	1.15	1.6
22	I feel competent about managing my time when I write down my appointments.	-0.48	0.91	-1.1

**Table 3:** Rasch analysis results on all 30 items together, the three subscales, and for tentative subscales with only fitting items.

	Item fit	PCA^a^ Eigenvalue in first contrast	Local independence largest interitem correlation	Item reliability	Person reliability	Number of strata	Mean person measure	Item DIF between male and female	Item DIF between different educational levels
ATMS 30 items	4 item misfit (no. 4, 10, 27, 28)	4.5	0.69	0.98	0.83	3	0.47		
Time management subscale 11 items	1 item misfit (no. 10)	2.1	0.20	0.96	0.77	2	1.45	No	No
Time management^∗∗^ subscale 10 items	No misfit	1.9	0.23	0.96	0.75	2	1.39	No	No
Organization & planning subscale 11 items	1 item misfit (no. 28)	1.9	0.23	0.98	0.70	2	0.32	No	No
Organization & planning subscale 10 items^∗∗^	No misfit	2.0	0.22	0.97	0.70	2	0.56	No	No
Regulation of emotions subscale 5 items	No misfit	1.6	0.01	0.98	0.38	1	0.02	No	No

^a^PCA: principal component analysis of residuals. ^∗∗^Tentative subscales with only fitting items included. These are not part of the final result.

**Table 4:** Conversion table from sum score to ATMS units.

Sum score	ATMS units
*Time management subscale*	
11	0
12	10
13	16
14	20
15	23
16	26
17	28
18	30
19	32
20	34
21	35
22	37
23	39
24	41
25	42
26	44
26	46
28	48
29	50
30	52
31	54
32	56
33	58
34	61
35	63
36	65
37	68
38	70
39	73
40	76
41	79
42	83
43	90
44	100
*Organization and planning subscale*	
11	0
12	12
13	19
14	24
15	27
16	30
17	33
18	35
19	37
20	39
21	41
22	43
23	45
24	46
25	48
26	50
26	51
28	53
29	54
30	56
31	57
32	59
33	60
34	62
35	64
36	66
37	67
38	69
39	72
40	74
41	78
42	82
43	89
44	100
*Regulation of emotions subscale*	
5	0
6	13
7	22
8	27
9	32
10	37
11	41
12	45
13	50
14	54
15	59
16	64
17	70
18	77
19	86
20	100

## Data Availability

The data collected for this study is not available for public access due to ethical considerations.
